# Occupational Therapy for Establishing a Morning Routine to Prevent Delirium After Hip Fracture Surgery: A Randomised Controlled Feasibility Study

**DOI:** 10.1111/psyg.70072

**Published:** 2025-07-21

**Authors:** Tomoko Kamimura, Keita Tomii

**Affiliations:** ^1^ School of Health Sciences Shinshu University Matsumoto Nagano Japan; ^2^ Department of Rehabilitation Aizawa Hospital Matsumoto Nagano Japan

**Keywords:** delirium, hip fractures, non‐pharmacological interventions, occupational therapy, older patients, prevention

## Abstract

**Background:**

Older patients are at a greater risk of developing postoperative delirium following hip fractures. Thus, multicomponent, non‐pharmacological interventions are recommended for preventing delirium. However, there is no consensus on the specific components that should be included in these interventions. Therefore, we developed an occupational therapy to establish a morning routine (OT‐EMR), incorporating the three components recommended in a Cochrane review. This study aimed to assess the feasibility of this program in patients aged ≥ 80 years following acute hip fracture surgery.

**Methods:**

This randomised controlled feasibility trial compared the outcomes of usual occupational therapy with those of OT‐EMR, which included daily interventions to support independence in activities of daily living each morning and group therapy sessions. Both groups received standard care, including early mobilisation, nutritional regulation, and other standard measures. The primary outcomes were delirium occurrence within 14 postoperative days and quality of life (QOL) on postoperative days 7 and 14.

**Results:**

Forty‐nine patients were enrolled (recruitment rate: 51.0%), and 45 patients (mean age: 89.2 years, standard deviation: 5.1) completed the study. The retention rate of the OT‐EMR was 88.0%. The respective incidence of delirium and the median duration of delirium were 45.5% and 1.5 days in the OT‐EMR group, compared with 56.5% and 3.0 days in the control group. The respective mean QOL values on days 7 and 14 were 0.570 and 0.612 in the OT‐EMR group, compared with 0.475 and 0.560 in the control group. Effect sizes were small across all outcomes.

**Conclusions:**

Implementation of OT‐EMR was feasible, but its effects on delirium incidence and QOL were small. Further improvements in the program implementation and research methods are essential for study continuation.

**Trail Registration:** University Hospital Medical Information Network Clinical Trials Registry number: UMIN000055541.

## 1 Introduction

Hip fractures are more common with increasing age, and the global incidence of new cases in patients aged 80 years and older was estimated to be 4.11 million (95% uncertainty interval, 2.97–5.62) in 2019 [1]. Postoperative delirium is a major complication in older patients with hip fractures, with a prevalence as high as approximately 50% [2, 3]. In these patients, delirium is associated with extended hospital stays [4, 5], increased healthcare costs [4], and high mortality rates [6]. Consequently, its prevention and management are critical [7, 8].

Multicomponent, non‐pharmacological interventions are recommended as evidence‐based strategies for preventing delirium [9, 10, 11, 12]. However, there is no consensus on the specific components that should be included in these interventions. A Cochrane review identified 12 components, highlighting sleep hygiene, cognitive stimulation, and re‐orientation as effective measures in reducing the risk of delirium, and recommended their inclusion in prevention programs [11]. Based on this evidence, the authors developed a delirium prevention intervention called occupational therapy to establish a morning routine (OT‐EMR), which integrates these three components. The intervention involves establishing a daily morning routine to promote sleep hygiene, providing guidance on morning activities of daily living (ADLs) (such as toileting and grooming) from an occupational therapist, and engaging patients in group therapy to facilitate cognitive stimulation and reality orientation.

The rationale for developing this programme is two‐fold: (1) establishing a morning routine may help mitigate sleep disturbances [13], and sleep promotion interventions are suggested to aid in preventing postoperative delirium [14]; and (2) cognitive stimulation and re‐orientation, particularly in group settings, have demonstrated promising effects on cognitive function and quality of life (QOL) in cognitively impaired individuals [15, 16, 17].

However, postoperative group therapy in acute hospitals is not common in clinical practice due to the difficulty in securing continuous group therapy participants along with postoperative management that requires individualized attention to patients. Moreover, to our best knowledge, no studies have evaluated its demand or potential efficacy. Therefore, in this study, we aimed to assess the feasibility of the OT‐EMR program as a precursor to an effectiveness study. We hypothesized that OT‐EMR after hip fracture surgery in older patients would be feasible in acute hospitals and may prevent delirium and improve QOL.

## 2 Methods

### 2.1 Study Design and Participants

This randomised controlled feasibility trial compared standard rehabilitation programmes with a specific programme incorporating OT‐EMR at Aizawa Hospital in Japan. The inclusion criteria were (1) age ≥ 80 years and undergoing surgery for acute hip fractures; and (2) ability to participate in group therapy, defined as (2‐1) the absence of trauma or fractures other than a hip fracture, (2) no coexisting illnesses or disabilities impeding participation, and (2, 3) the ability to leave the bed based on the criteria of the Japanese Society for Early Mobilisation (JSEM) [18]. Patients admitted to non‐orthopaedic wards after surgery were excluded before allocation. Additionally, patients in the OT‐EMR group who were unable to participate in group therapy for more than 3 days during the period were excluded from the analysis after allocation.

Patients or their legal representatives provided written informed consent to participate. The Shinshu University Ethics Committee approved the study protocol (approval number: 6013).

### 2.2 Intervention

Patients in the intervention group participated in the OT‐EMR programme, starting at 9:00 a.m. daily from the third to the fourteenth postoperative day. During the first hour, occupational therapists individually assisted patients in performing ADLs, such as toileting and grooming, as independently as possible. Then, they formed groups and led group activities, such as self‐introduction, radio callisthenics, balloon volleyball, and bowling. These were followed by 2 h of static activities facilitated by care workers, such as block‐building games, quizzes, music appreciation, and creative activities related to seasonal events. Patients with hearing impairments were provided cartilage conduction hearing aids to facilitate participation. Participation time in group sessions was adjusted daily on the basis of each patient's level of fatigue. All group therapies incorporated enjoyable activities designed to stimulate concentration, memory, thinking, and orientation.

Patients in the control group received standard occupational therapy (OT), consisting of individualized ADL exercises for 1 h in the afternoon instead of OT‐EMR during the same period. Their participation in the care worker‐led group sessions was voluntary.

All patients in both groups received daily physical therapy from the first postoperative day until discharge, standard OT on postoperative days 1 and 2, and daily OT from postoperative day 15 until discharge. During hospitalization, nutritional and hydration regulation, pain management, and consultation with a psychiatrist were provided as needed.

### 2.3 Data Collection

Participants were assigned to the intervention and control groups using a stratified block method. The allocation adjustment factor was based on the severity of cognitive impairment (assessed using the Mini‐Mental State Examination‐Japanese [MMSE‐J] ≤ 20 vs. MMSE‐J > 20). The MMSE‐J was administered preoperatively by an occupational therapist or within 1 day postoperatively if the preoperative evaluation was not feasible due to emergency surgery.

The Confusion Assessment Method (CAM) [19] was used to evaluate postoperative delirium. Occupational therapists and nurses, trained in advance on delirium and CAM, conducted the daily assessments. The incidence and duration of delirium were measured during the 14‐day postoperative period.

QOL was assessed by an occupational therapist on postoperative days 7 and 14 using the Euro Qol 5 Dimensions 5‐Level (EQ‐5D‐5L) questionnaire, with utility values calculated.

Participant characteristics included age, sex, pre‐fracture ambulation status (Functional Ambulation Categories [20]), place of residence, presurgery albumin levels, comorbidity burden (Functional Comorbidity Index [21]), American Society of Anesthesiologists (ASA) physical status classification [22], fracture type, surgical delays exceeding 2 days, surgical treatment, and length of hospital stay.

Recruitment, retention rate, and limited efficacy were assessed for feasibility. Limited efficacy testing was conducted to estimate effect sizes.

### 2.4 Statistical Analysis

Categorical data are presented as frequencies and percentages. Continuous data with normal distributions are reported as means and standard deviations (SDs), whereas non‐normally distributed data are reported as medians and interquartile ranges (IQRs).

For group comparisons, the chi‐square test or Fisher exact test was applied to categorical data, *t*‐test to normally distributed continuous data, and Mann–Whitney *U* test to non‐normally distributed continuous data. Statistical significance was defined as *p* < 0.05. Effect sizes were calculated as follows: *ϕ* for the chi‐square test, Cohen *d* for the *t*‐test, and *r* (after *Z*‐transformation) for the Mann–Whitney *U* test. Thresholds for effect sizes were defined as follows: for *ϕ* and *r*, 0.1 (small), 0.3 (medium), and 0.5 (large); and for Cohen *d*, 0.2 (small), 0.5 (medium), and 0.8 (large).

All analyses were performed using SPSS Statistics for Windows (version 29.0; IBM Corp., Armonk, NY, USA).

## 3 Results

**FIGURE 1:**
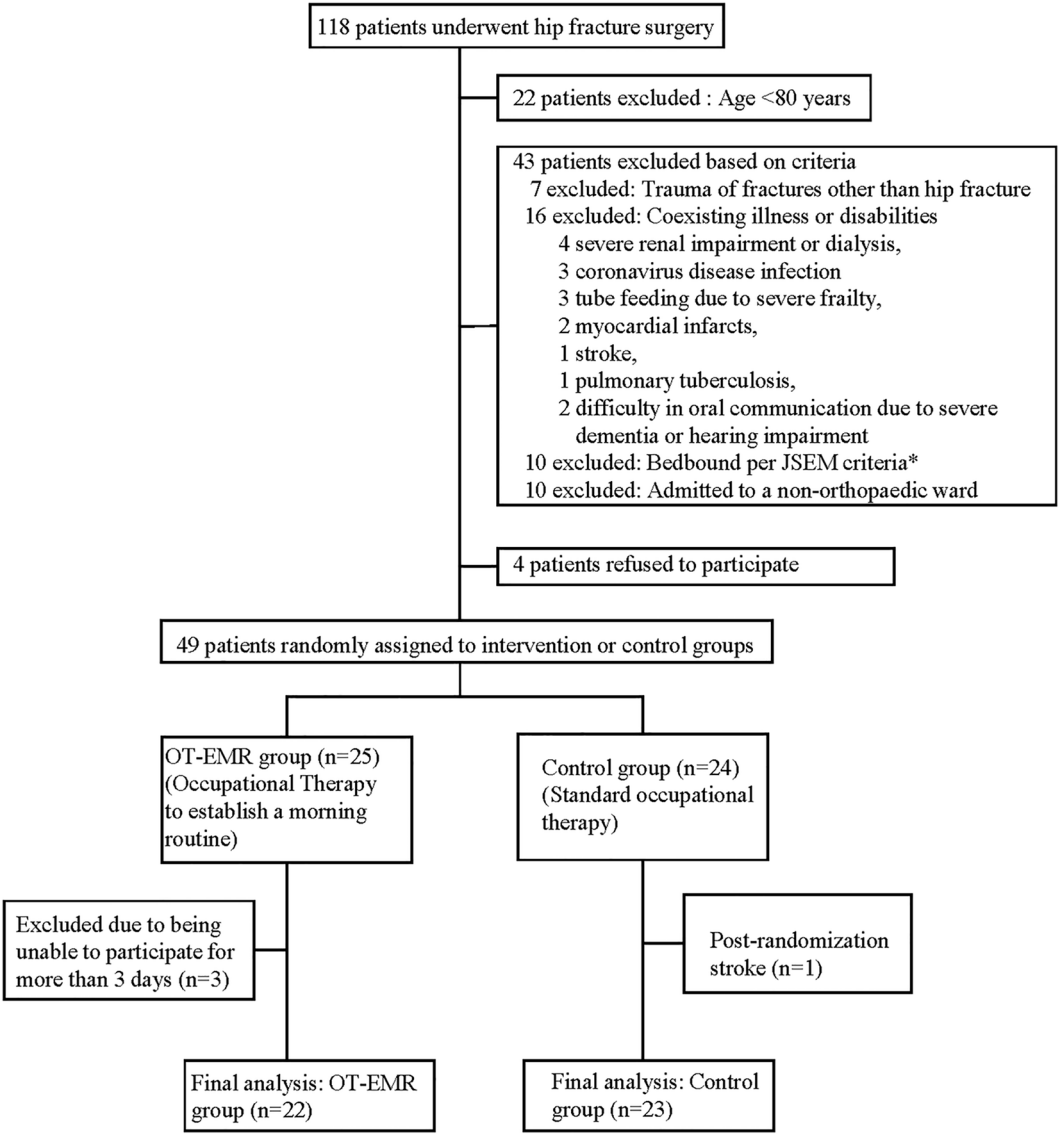
Flowchart of participant eligibility and inclusion. (*) The Japan Society for Early Mobilization (JSEM) lists five conditions in which it is contraindicated to leave the bed and seven conditions in which it is necessary to carefully consider whether to leave the bed or not. The conditions applied in this study were as follows: uncontrolled fatal arrhythmia, fever of 38°C or higher, resting heart rate of 50 beats/min or lower or 120 beats/min or higher, resting systolic blood pressure of 80 mmHg or lower, and abnormal breathing even at rest. COVID‐19, coronavirus disease; OT‐EMR, Occupational therapy to establish a morning routine.

A total of 118 patients underwent acute hip fracture surgery between December 1, 2023, and March 22, 2024. Ninety‐six patients were aged ≥ 80 years, and 49 patients were included in the study (recruitment rate, 51.0%) (Figure 1). The following 43 patients were excluded before allocation according to the aforementioned criteria: seven patients with trauma or fracture other than hip fracture, 16 patients with illness or disability that would impede their participation in the group therapy, 10 patients who could not leave the bed according to the JSEM criteria, and 10 patients admitted to another ward because the orthopaedic ward was full. The illnesses and disabilities that were considered reasons for the exclusion of 16 patients included severe renal impairment or dialysis, coronavirus disease infection, tube feeding due to severe frailty, myocardial infarcts, stroke, pulmonary tuberculosis, and difficulty in oral communication due to severe dementia or hearing impairment. Of the 53 patients who met the criteria, four refused to participate (consent rate, 92.5%). Of the remaining 49 patients, three withdrew from the OT‐EMR group and one from the control group, leaving 22 and 23 patients for analysis, respectively. The retention rate for the OT‐EMR group was 88.0%. The three participants who withdrew from the study did so because they were unable to participate in group therapy for more than 3 days during the study period due to the nursing care or treatment.

Table 1 presents the characteristics of the 45 participants included in the analysis. The mean age was 89.2 years (SD = 5.1), and most participants were female (*n* = 39, 86.7%). The mean MMSE‐J score was 15.8 (SD = 7.8), with 11 individuals (24.4%) scoring 0–10 (severe cognitive impairment [23]), 21 (46.7%) scoring 11–20 (moderate cognitive impairment [23]), and 13 (28.9%) scoring 21–29 (mild or questionable impairment [23]). Most participants could walk independently on a level surface according to Functional Ambulation Categories 4–5 before their fractures (*n* = 40, 88.9%). Thirty‐two participants (71.1%) lived at home. Most participants had no or few comorbidities according to Functional Comorbidity Indexes 0–1 (*n* = 34, 75.6%); their preoperative physical status was classified as mild systemic disease according to the ASA classification (*n* = 39, 86.7%), and the median length of hospital stay was 22.0 days (IQR = 7.0). No significant differences in the measured characteristics were observed between the OT‐EMR and control groups.

**TABLE 1:** Participants' characteristics.

	All participants (*n* = 45)	OT‐EMR group (*n* = 22)	Control group (*n* = 23)	*p*‐value
Age (years)	89.2 ± 5.1	89.4 ± 4.8	89.1 ± 5.4	0.879
Sex, female	39 (86.7%)	18 (81.8%)	21 (91.3%)	0.414
MMSE‐J score	15.8 ± 7.8	16.9 ± 8.5	14.9 ± 7.2	0.399
MMSE‐J				
0–10 (severe CI)	11 (24.4%)	5 (22.7%)	6 (26.1%)	0.908
11–20 (moderate CI)	21 (46.7%)	10 (45.5%)	11 (47.8%)	
21–29 (mild and questionable CI)	13 (28.9%)	7 (31.8%)	6 (26.1%)	
FAC				
1–3	5 (11.1%)	3 (13.6%)	2 (8.7%)	0.665
4–5	40 (88.9%)	19 (86.4%)	21 (91.3%)	
Residence before fracture, home	32 (71.1%)	15 (68.2%)	17 (73.9%)	0.672
Albumin level before surgery (g/dL)	3.31 ± 0.34	3.28 ± 0.32	3.34 ± 0.37	0.522
FCI				
0–1	34 (75.6%)	16 (72.7%)	18 (78.3%)	0.738
2–3	11 (24.4%)	6 (27.3%)	5 (21.7%)	
ASA classification				
II	39 (86.7%)	17 (77.3%)	22 (95.7%)	0.096
III	6 (13.3%)	5 (22.7%)	1 (4.3%)	
Fracture type				
Femoral neck	27 (60.0%)	14 (63.6%)	13 (56.5%)	0.626
Intertrochanteric	18 (40.0%)	8 (36.4%)	10 (43.5%)	
Surgical delay of > 2 days	6 (13.3%)	5 (22.7%)	1 (4.3%)	0.096
Surgical treatment				
BHA	24 (53.3%)	12 (54.5%)	12 (52.2%)	0.873
Intramedullary nail	21 (46.7%)	10 (45.5%)	11 (47.8%)	
Length of hospital stay (days)^a^	22.0 [7.0]	21.0 [5.0]	23.0 [7]	0.412

*Note:* Data are presented as number (percentage), mean ± standard deviation, or median [interquartile range].

Abbreviations: ASA, American Society of Anesthesiologists; BHA, bipolar hip arthroplasty; CI, cognitive impairment; FAC, Functional Ambulation Categories; FCI, Functional Comorbidity Index; MMSE‐J, Mini‐Mental State Examination‐Japanese; OT‐EMR, occupational therapy to establish a morning routine.

^a^ The Mann–Whitney *U* test was performed for length of stay, whereas the *t*‐test was used for all other variables.

The care worker‐led group sessions were consistently attended by two to five patients throughout the study period.

Table 2 shows the results of the limited efficacy testing. The incidence of delirium and the median number of delirium days were 45.5% and 1.5 days (IQR = 13.0), respectively, in the OT‐EMR group, compared with 56.5% and 3.0 days (IQR = 13.0) in the control group. The mean QOL utility values on days 7 and 14 were 0.570 (SD = 0.211) and 0.612 (SD = 0.149) in the OT‐EMR group, and 0.475 (SD = 0.243) and 0.560 (SD = 0.246) in the control group, respectively. These results were not significantly different between the groups. Effect sizes were small for all outcomes.

**TABLE 2:** Results of the limited efficacy testing.

	OT‐EMR group (*n* = 22)	Control group (*n* = 23)	Test statistic	*p*‐value	Effect size	
Incidence of delirium in 14 days	10 (45.5%)	13 (56.5%)	*χ* ^2^	0.458	*Φ* = 0.111	Small
Days of delirium occurrence in 14 days	1.5 [13.0]	3.0 [13.0]	Mann–Whitney *U*	0.784	*r* = 0.274	Small
QOL utility value						
Postoperative day 7	0.570 ± 0.211^a^	0.475 ± 0.243	*t*	0.177	Cohen *d* = 0.415	Small
Postoperative day 14	0.612 ± 0.149	0.560 ± 0.246	*t*	0.395	Cohen *d* = 0.254	Small

*Note:* Data are presented as number (percentage), median [interquartile range], or mean ± standard deviation.

Abbreviation: OT‐EMR, occupational therapy to establish a morning routine.

^a^ Only the QOL utility value on postoperative day 7 in the OT‐EMR group has missing data.

## 4 Discussion

This study's results indicate that the recruitment rate for OT‐EMR was approximately 50%, with both the consent and retention rates reaching approximately 90%. This suggests that OT‐EMR is acceptable and implementable, and it demonstrates good feasibility in these areas, partially confirming our hypothesis. To our best knowledge, this is the first study to demonstrate that a hospital performing approximately 100 hip fracture surgeries over 4 months can provide group therapy for two to five patients daily, all older than 80 years of age. This finding suggests that there may be a demand for such a program, even in acute hospital settings following surgery.

One reason for the good consent and retention rates, despite the participants' advanced age, may be the exclusion of those in poor postoperative physical condition. Therefore, most participants had fewer functional impairments and cumulative comorbidities before their fractures, which are predisposing factors for delirium [24].

However, in this study, the effect size of OT‐EMR on both the delirium incidence and patient QOL was small. One potential reason for this is that the control group also received comprehensive delirium prevention measures, including early mobilisation by physical therapists, regulation of nutrition and hydration, pain management, consultation with a psychiatrist, and standard OT with ADL exercises. Consequently, simply replacing standard OT with OT‐EMR may have provided limited additional effectiveness. Previous studies have suggested that OT in the postoperative period [25] and in the intensive care unit [26] positively affects delirium prevention, ADL performance, and cognitive function. In these studies, control groups received minimal occupational therapist involvement, whereas in this study, the control group received 1 h of daily OT. This higher baseline level of OT involvement may explain the smaller observed effect size when switching to OT‐EMR.

Another possible reason for the small effect could be issues with the implementation of the programme or the research methods. Previous studies have reported that delirium prevention measures should be initiated as early as possible [27], and that group therapy is most effective for older patients with mild to moderate cognitive impairment [15, 16, 17]. In this study, OT‐EMR was not started until postoperative day 3, and approximately 25% of participants had severe cognitive impairment because participants were not restricted on the basis of the severity of their cognitive impairment. In this study, these conditions were lenient owing to the nature of introducing a new programme into clinical practice; however, there is room for improvement in these areas. Further investigation is needed to determine whether the limited effectiveness was due to the inherent nature of OT‐EMR or whether improving these programme implementation and research methods could enhance the impact of OT‐EMR.

This study has some limitations. First, it was a feasibility study with a small sample size conducted at a single institution. Second, the differences in medication and other treatments provided during the observation period between the two groups were not examined. Additionally, some patients were excluded before allocation for hospital administrative reasons, such as being admitted to non‐orthopaedic wards post‐surgery and after allocation in which OT‐EMR could not be performed for more than 3 days during the period due to nursing care or other reasons, potentially introducing sample bias. Third, there was no diagnosis of delirium by a physician. Lastly, the MMSE‐J, CAM, and QOL assessments were conducted by the treating occupational therapists, raising the possibility of examiner bias. These issues should be addressed as much as possible before proceeding to a randomized controlled trial.

In conclusion, the OT‐EMR programme was feasible in this patient population; however, compared with standard OT, its effects on delirium incidence and patient QOL were small. For study continuation, improvements in programme implementation and research methods are necessary.

## Ethics Statement

The Shinshu University Ethics Committee approved the study protocol (approval number: 6013).

## Consent

Patients or their legal representatives provided written informed consent to participate.

## Conflicts of Interest

The authors declare no conflicts of interest.

## Data Availability

The data that support the findings of this study are available from the corresponding author upon reasonable request.
